# Renalase Peptide (RP220) Activates MAPK Signaling Pathways in Primary Mouse Skeletal Muscle Cells

**DOI:** 10.17912/micropub.biology.002195

**Published:** 2026-09-14

**Authors:** Katsuyuki Tokinoya, Yuri Kato, Kai Aoki, Kazuhiro Takekoshi

**Affiliations:** 1 College of Education, Yokohama National University, Yokohama, 14, Japan; 2 University of Tsukuba, Tsukuba, 08, Japan; 3 Niigata University of Health and Welfare, Niigata, 15, Japan

## Abstract

Renalase is a secreted protein that was initially discovered in the kidney and that is also expressed in skeletal muscle. Moreover, renalase peptide RP220 activates MAPK signaling in the kidneys. This study investigated the effects of the RP220 on skeletal muscle using primary cultured cells. Notably, RP220 treatment did not alter the myogenic differentiation phenotype; however, intracellular signaling analyses revealed significant phosphorylation of p38 and ERK, which are MAPK family members. These results indicate that renalase affects MAPK signaling, but its physiological or developmental effects on skeletal muscle are not yet known.

**Figure 1. MAPKs signaling in primary mice myotubes treated with RP220:** (A) MAPK signaling in myotubes treated either with human- or mouse- derived RP220. (B) Immunofluorescence analysis of differentiation at day 5 (n = 4). The fusion index of the myotubes was calculated as the percentage of nuclei incorporated in the myotubes (MHC: green) relative to the total DAPI count (blue). Scale bar, 200 μm. (C) Time course experiment showing MAPK family member modifications in myotubes treated with RP220. MHC, myosin heavy chain; Scr, scramble control; hRP220, human RP220; mRP220, mouse RP220. Values represent the mean ± SEM (n = 3 or n = 4). * *p* < 0.01, ** *p* < 0.001 vs. Scr or 0 min.

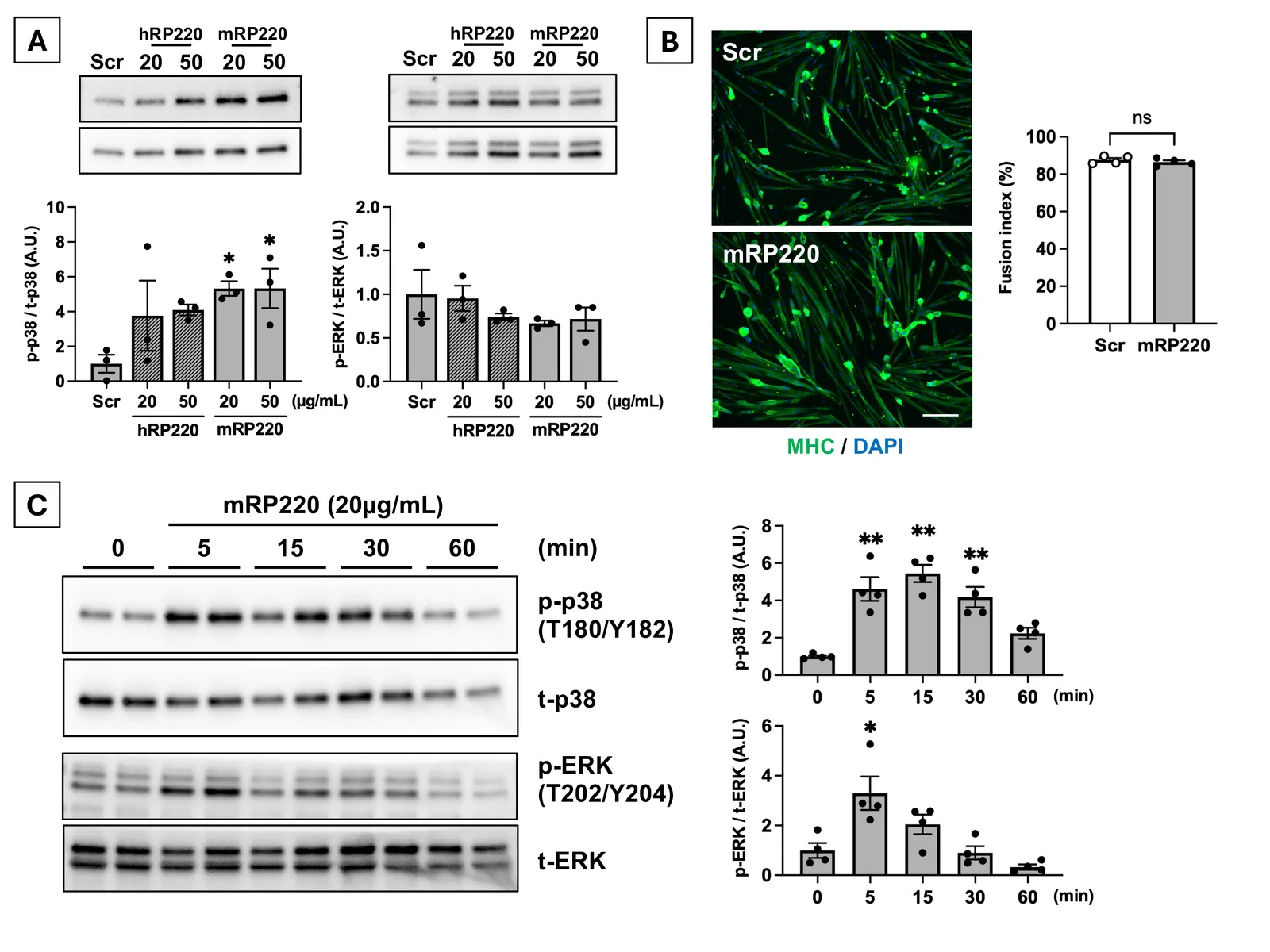



## Description

Skeletal muscle serves not only as a locomotor organ but also as an endocrine organ that secretes bioactive substances known as myokines. Renalase is expressed in skeletal muscle, and both its expression and blood levels increase following acute exercise (Tokinoya et al., 2018, 2020), suggesting its potential role as a myokine. Previous studies in the heart and kidney have reported that renalase, specifically through its 20-amino acid peptide RP220, which starts at residue 220, activates intracellular signaling pathways, such as MAPK (p38 and ERK) pathways, via its receptor (Wang et al., 2015). However, the detailed mechanism of action within skeletal muscle cells remains unclear. This study aimed to clarify the effects of RP220 on differentiation and MAPK signaling in primary mouse skeletal muscle cells (myotubes). First, we analyzed the response of intracellular signaling pathways to RP220 in differentiated myotubes to determine optimal treatment conditions. In a 30-min treatment, differentiated myotubes were treated with human- or mouse-derived RP220 at 20 or 50 µg/mL. Separate one-way ANOVAs were performed for human- and mouse-derived RP220. Compared with the scramble (Scr) control group, mouse-derived RP220 significantly increased p38 phosphorylation at both 20 and 50 µg/mL (both *p* < 0.05), whereas human-derived RP220 did not significantly alter p38 phosphorylation at either concentration. ERK phosphorylation remained unchanged ( Figure 1A ). A time-course experiment (0, 5, 15, 30, and 60 min) using mouse RP220 further revealed that p38 phosphorylation was significantly increased at 5, 15, and 30 min posttreatment, whereas that of ERK was significantly elevated only at min 5 posttreatment ( Figure 1C ). Based on these responses, we subsequently evaluated the effect of 20 µg/mL RP220 in the differentiation of myoblasts into myotubes. Notably, myosin heavy chain (MHC) immunocytochemistry analyses showed no significant differences in the differentiation potential of the RP220-treated and Scr control groups ( Figure 1B ).

 p38 phosphorylation by RP220 was consistent with that previously reported in other organs and is expected to involve pathways related to cell proliferation and differentiation. Additionally, since ERK regulates the expression of specific genes that promote the slow-twitch phenotype in skeletal muscle, the ERK phosphorylation pattern observed here may indicate a specific role for renalase in fiber-type regulation. In conclusion, while RP220 significantly phosphorylates p38 and ERK in myotubes, the current dose appears insufficient to alter myotube differentiation morphology. Further studies are required to determine the optimal concentration and timing for long-term chronic treatment throughout the differentiation period of these syncytial cells.

## Methods

The experiments were conducted with the approval of the Animal Experiment Committee of the University of Tsukuba (Approval Number: 24-158).

Male C57BL/6J mice (8–12 weeks old) were used for this study. Extensor digitorum longus (EDL) muscles were isolated and digested with type I collagenase to isolate single muscle fibers, as described previously (Kato et al., 2026). Fibers were seeded on Matrigel-coated dishes and cultured in growth media (DMEM with 30% FBS, 1% GlutaMAX, 1% chicken embryo extract, 1% penicillin–streptomycin, and 10 ng/mL bFGF) to collect myoblasts. For differentiation, myoblasts were switched to a differentiation medium (DMEM with 5% HS and 1% penicillin–streptomycin) for 5 days.

The comparison between the human and mouse RP220-treated (AAs: human, CIRFVSIDNKKRNIESSEIG; mouse, CICFISIDNKKRNIESSECG) and Scr control (AAs: CSKRIFKVISSIEDNNERG) groups was performed at 20 and 50 µg/mL for 30 min, and time-course experiments using 20 µg/mL mouse RP220 were performed over a 0–60 min period. For differentiation experiments, myotubes were cultured for 5 days in medium supplementedwith either mouse RP220 or Scr.

For MAPK signaling analysis, myotubes differentiated without RP220 were preincubated in either serum-free medium for 24 h or in 1% HS medium for 16 h prior to the addition of RP220. On day 5, myotubes were either fixed with 4% PFA for MHC immunofluorescence and DAPI counterstaining or lysed with lysis buffer (50 mM Tris-HCl pH 7.4, 150 mM NaCl, 1% NP-40, 1 mM EDTA) for western blot analysis. The following antibodies were used: anti-myosin, heavy chain (R&D Systems, Cat# MAB4470, clone MF20), Alexa Fluor 488 goat anti-mouse IgG (Jackson ImmunoResearch, Cat# 115-545-003), anti-phospho-p44/42 MAPK (ERK1/2, Thr202/Tyr204) (Cell Signaling Technology, Cat# 4370), anti-p44/42 MAPK (ERK1/2) (Cell Signaling Technology, Cat# 4695), anti-phospho-p38 MAPK (Thr180/Tyr182) (Cell Signaling Technology, Cat# 4511), anti-p38 MAPK (Cell Signaling Technology, Cat# 9212), anti-rabbit IgG, HRP-linked antibody (Cell Signaling Technology, Cat# 7074). Phosphorylated p38 MAPK and ERK1/2 levels were normalized to their corresponding total p38 MAPK and total ERK1/2 levels, respectively.

Statistical analyses were performed using GraphPad Prism, employing Student’s *t* -tests or one-way ANOVA with Dunnett’s post-hoc tests.

## References

[R1] Kato Yuri, Tokinoya Katsuyuki, Aoki Kai, Takekoshi Kazuhiro (2026). Renalase knockdown inhibits proliferation of mouse satellite cells. Molecular Biology Reports.

[R2] Tokinoya Katsuyuki, Shiromoto Jun, Sugasawa Takehito, Yoshida Yasuko, Aoki Kai, Nakagawa Yoshimi, Ohmori Hajime, Takekoshi Kazuhiro (2018). Influence of acute exercise on renalase and its regulatory mechanism. Life Sciences.

[R3] Tokinoya Katsuyuki, Yoshida Yasuko, Sugasawa Takehito, Takekoshi Kazuhiro (2020). Moderate‐intensity exercise increases renalase levels in the blood and skeletal muscle of rats. FEBS Open Bio.

[R4] Wang Ling, Velazquez Heino, Chang John, Safirstein Robert, Desir Gary V. (2015). Identification of a Receptor for Extracellular Renalase. PLOS ONE.

